# Medical News and Miscellany

**Published:** 1899-05

**Authors:** 


### Medical News and Miscellany.

**Dr. T. Belsher** has removed from Saddler to Whitesboro, Texas.

**Compulsory Vaccination** in Texas is a pressing necessity. Mexico has adopted it.

**Dr. J. C. Jones,** of Gonzales, Texas, Hood's Chief Surgeon during the Civil War, has been appointed Surgeon-General, Texas Division, United Confederate Veterans.

**A Sad Commentary.**—"The great State of Texas keeps a system of vital statistics for its pigs, but none for its people."—M. M. Smith, M. D., Annual Oration.

**Alumni of Jefferson Medical Cellege** everywhere, are requested to communicate with the editors of "The Jeffersonian," Jefferson Medical College, Philadelphia.

**The Paper on Tonsilitis** in our last issue was by Dr. A. Nowlin, Hutto, Texas. His name was omitted inadvertently, for which we tender the Doctor our apologies.

**Change of Locations.**—Dr. H. W. Robertson from Sublime, Texas, to Waelder. Dr. F. A. Young from Navasota to Sublime. Dr. S. C. Gage from Gatesville to Ennis, Texas.

**San Antonio** has recently put in a $500,000 sewer system, and Dr. Frank Paschall, the newly elected Health Officer on the Reform ticket, is vigorously pushing a most commendable sanitary reform.

**For Sale or Trade.**—A small property and a good practice in a rich country near the coast, central part; will sell on easy terms or trade for a place in West Texas or New Mexico. Address S. E. E., care Texas Medical Journal, Austin, Texas.

**Dr. Hugh Young,** of the Johns-Hopkins Hospital Medical College, attended the Texas State Medical Association meeting at San Antonio, and contributed a paper on "Operation for Enlarged Prostate." He is a son of General Young, C. S. A., of San Antonio.

---

**Send Us Some Copies** of our January number. We will credit each sender with one month on his subscription for each copy sent. The demand for this edition was so great that we let go our file numbers rather than disappoint any one. The attraction was "Sanitary Needs of Texas."

---

**We are in Receipt** of a beautiful lithographed pamphlet from the ever enterprising Mr. Henry of Louisville, who in a letter informs us of his now being principal owner of the famous French Lick Springs of Indiana, capacity 500 guests. The celebrated Spa-Pluto, America's aperient he will introduce at once through the medical press as the most saline hydragogue eliminant and intestinal antiseptic akin to Carlsbad, without the accompanying nausea and thirst.

---

**Vote of Thanks to Dr. Blunt.**—The citizens of Laredo, Texas, adopted a set of most complimentary resolutions expressive of their gratitude to and confidence in State Health Officer Blunt, and congratulating the State of Texas upon having for State Health Officer a physician of such distinguished skill, energy and courage. Dr. Blunt has been in the quarantine business nearly all of his professional life and certainly understands the process of "stamping out." Quarantine against Laredo was raised on May 1.

